# Rheological Properties and Microscopic Mechanisms of Composite-Modified Asphalt with Direct Coal Liquefaction Residue

**DOI:** 10.3390/polym18101192

**Published:** 2026-05-13

**Authors:** Yongxiang Li, Chaoyang Guo, Shizhong Mi, Xuliang Zhang, Jinbo Bai, Yongjie Jia, Hongyin Yu, Jing Li

**Affiliations:** 1 College of Energy and Transportation Engineering, Inner Mongolia Agricultural University, Hohhot 010018, China; 15226806131@163.com (Y.J.); yuhongyin5289@163.com (H.Y.); li1728401678@163.com (J.L.); 2 Inner Mongolia Transportation Group Co., Ltd., Hohhot 010010, China; 17615816029@163.com (C.G.); 17719759926@163.com (S.M.); 13665761666@163.com (X.Z.); zhangliqing0206@163.com (J.B.)

**Keywords:** direct coal liquefaction residue, composite modification, rheological properties, low-temperature performance, microscopic mechanism

## Abstract

To enhance the overall performance of direct coal liquefaction residue (DCLR)-modified asphalt, particularly its low-temperature cracking resistance, SBS and aromatic oil were employed for composite modification. Nine composite-modified asphalt formulations were prepared based on an orthogonal experimental design. High-and low-temperature rheological properties and microstructure of all modified asphalts were systematically evaluated using a dynamic shear rheometer (DSR), a bending beam rheometer (BBR), Fourier-transform infrared spectroscopy (FTIR), and scanning electron microscopy (SEM). The results indicate that composite modification significantly enhanced the high-temperature performance of the asphalt. Modified asphalt labeled as Sample No. 9 (9% DCLR, 4% SBS, and 6% aromatic oil) demonstrated the minimal non-recoverable creep compliance ($J_{nr}$) value of 0.58 kPa^−1^ at 64 °C, indicating a 78.6% decrease relative to the matrix asphalt. In terms of low-temperature performance, Sample No. 3 satisfied the Superpave cracking resistance criterion, exhibiting a creep rate (m-value) of 0.312 at −12 °C. It was revealed by FTIR analysis that the interaction between the composite modifier and the base asphalt was mainly physical blending, and no new functional groups were generated either before or after aging. The improvement in performance was attributed to the physical compatibility and structural reorganization among the components. Microstructural analysis revealed that the uniform dispersion of modifiers in matrix asphalt and the subsequent formation of a dense micelle structure after aging contributed to the enhanced macroscopic performance. This study provides theoretical and technical support for the high-value application of DCLR in asphalt pavements.

## 1. Introduction

Direct coal liquefaction technology offers a clean utilization approach for efficiently transforming coal into light fuels and high-value-added chemical products, significantly contributing to the structural adjustment of China’s coal industry [1,2,3]. This process generates direct coal liquefaction residue (DCLR), which accounts for approximately 30% of the mass of the raw coal feedstock [4]. Current primary utilization methods for DCLR include hydrogenation liquefaction [5], gasification [6], pyrolysis and coking [7], use as boiler fuel [8,9], and the preparation of carbon-based materials [10,11,12]. From the perspectives of enhancing the overall economics of the coal liquefaction process, achieving comprehensive resource utilization, and environmental protection, the efficient conversion and utilization of DCLR is therefore imperative [13].

Research indicates that the dosage of DCLR significantly influences the properties of the modified asphalt. Generally, as the DCLR content increases, the high-temperature performance of the asphalt improves progressively, while its low-temperature ductility declines accordingly [14,15,16]. Regarding preparation technology, DCLR is typically ground into a fine powder before being blended into the base asphalt to promote its dispersion and compatibility within the asphalt matrix [17]. Comparative analyses show that DCLR can partially replace natural asphalt (e.g., TLA) as a modifier, often requiring a lower dosage; its performance is comparable to SBS-modified asphalt at a certain content (e.g., 5%), and it exhibits superior adhesion and crack resistance compared to conventional base asphalt [18,19]. To further enhance the overall pavement performance, composite modification has become an important strategy: the combination of DCLR and SBS can synergistically improve both the high-and low-temperature properties of asphalt [20]; when compounded with crumb rubber, DCLR primarily enhances high-temperature stability, while the rubber provides elasticity, jointly compensating for deficiencies in low-temperature performance [21]. Furthermore, the incorporation of compatibilizers or plasticizers can also effectively tune the properties of DCLR-modified asphalt [22]. In practical engineering applications, studies have confirmed that DCLR-modified asphalt mixtures meet specification requirements for high-temperature stability, moisture susceptibility, and overall pavement performance [23], demonstrating promising potential for engineering applications.

From a rheological perspective, direct coal liquefaction residue exhibits characteristics typical of a non-Newtonian pseudoplastic fluid, with its viscosity gradually decreasing as temperature rises; within a higher temperature range, its flow behavior approximates that of a Newtonian fluid [24]. Studies have shown that DCLR, as a modifier, can significantly enhance the permanent deformation resistance of asphalt and effectively reduce its temperature susceptibility [25,26]. Evaluations based on indicators such as non-recoverable creep compliance $J_{nr}$ further corroborate the role of DCLR in improving the high-temperature performance of asphalt. However, existing research findings regarding the impact of DCLR on the fatigue performance of asphalt are inconsistent. Some studies indicate that the fatigue performance of DCLR-modified asphalt surpasses that of base asphalt, with improvements in rutting and fatigue resistance under high-and intermediate-temperature conditions verifiable through frequency sweep, multiple stress creep recovery (MSCR), and linear amplitude sweep (LAS) tests [27,28]. Conversely, other studies, based on the analysis of dynamic shear rheological parameters, suggest that DCLR may exert a certain negative influence on the fatigue performance of asphalt [29,30]. These discrepancies may be attributed to differences in testing conditions, evaluation systems, and modification formulations, and the underlying mechanisms warrant further systematic investigation.

DCLR is fundamentally a complex mixture, primarily composed of unreacted organic carbon, partially liquefied heavy oil, intermediate inorganic minerals, and residual liquefaction catalysts [31]. This material is characterized by high ash, carbon, sulfur, and heteroatom contents in its chemical composition. Its typical components include heavy oil, asphaltenes, pre-asphaltenes, and tetrahydrofuran-insoluble solids [5,32,33,34,35]. Research indicates that the viscosity of DCLR is significantly influenced by its carbon and ash content, while the physicochemical properties of its contained heavy oil are similar to those of the organic matter in coal [36]. Structurally, the heavy oil and asphaltenes within the residue are primarily composed of two-to three-ring aromatics, with aromatic rings bearing alkyl side chains containing approximately nine to ten carbon atoms. The asphaltenes present a structure with high aromaticity and carbon content, consisting of polycyclic aromatic hydrocarbons and their alkyl substituents [37,38]. Furthermore, the variety and content of aromatic substances in DCLR are significantly higher than in other components. Its macromolecular units predominantly exhibit fused-ring structures, resulting in an overall appearance and high viscosity similar to petroleum residues, albeit with a more complex chemical composition. Synergistic interactions exist among its various components, endowing DCLR with the potential to serve as an asphalt modifier [39]. Further studies demonstrate that the incorporation of DCLR adsorbs the saturates and aromatics in asphalt, thereby increasing the proportion of resins and asphaltenes, expanding the range of large molecular domains within the asphalt, and increasing its polydispersity index (PDI) [40]. Microscopic characterization techniques, such as Fourier-transform infrared spectroscopy (FTIR), atomic force microscopy (AFM), saturates–aromatics–resins–asphaltenes (SARA) analysis, and gel permeation chromatography (GPC), can provide in-depth insights into the mechanistic role of DCLR within the asphalt system [41]. Coupled with scanning electron microscopy (SEM) and image-processing techniques, these methods allow for a direct evaluation of the improvement in the low-temperature performance of DCLR-modified asphalt achieved through the use of compatibilizers [42,43].

Although previous studies have indicated that DCLR can enhance the high-temperature performance of asphalt and that its combination with polymers such as SBS can improve the low-temperature performance, existing research has mostly focused on single-or two-component modification systems. A systematic investigation into the ternary composite system of DCLR–SBS–aromatic oil is still lacking. Furthermore, most studies have emphasized macroscopic performance evaluation without revealing the synergistic modification mechanism from the combined perspectives of rheology and microstructure. To this end, an orthogonal experimental design was adopted in this study, through which the effects of DCLR, SBS, and aromatic oil on the rheological properties of asphalt were systematically investigated for the first time. By means of dynamic shear rheometer (DSR), bending beam rheometer (BBR), FTIR, and SEM, the evolution mechanism of the high-and low-temperature performance of the ternary composite-modified asphalt was revealed at both the macroscopic and microscopic levels, aiming to provide a theoretical basis for the high-value utilization of DCLR.

## 2. Materials and Methods

### 2.1. Materials

In this study, the 90# grade A base asphalt produced by Donghai Brand was carefully selected. The results of its conventional property tests are presented in Table 1, and all relevant parameters fully meet the requirements of the specification. The DCLR, which consists of undecomposed heavy oil, asphaltenes, pre-asphaltenes, and tetrahydrofuran-insoluble matter, is characterized by the technical indicators listed in Table 2. A commonly used linear SBS modifier, YH-791, with a styrene–butadiene block ratio of 3:7, was utilized, and its basic properties are provided in Table 3. A 2#-38 aromatic oil was chosen as the compatibilizer for the composite-modified asphalt.

### 2.2. Preparation of DCLR-Modified Asphalt

**Figure 1:**
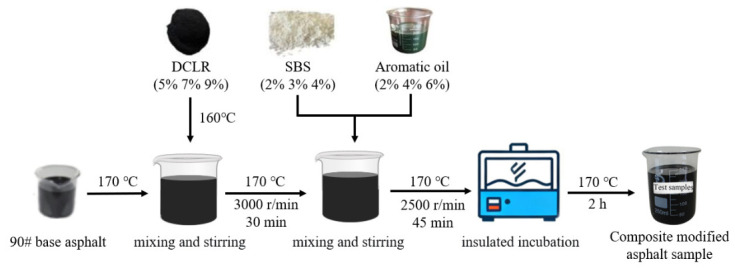
Flowchart of the preparation process of the composite-modified asphalt.

To enhance the low-temperature performance of DCLR-modified asphalt, a composite modification method incorporating SBS and aromatic oil was adopted in this research. An orthogonal experimental design with three factors and three levels was carried out based on the variables of DCLR, SBS, and aromatic oil (see Table 4 and Table 5). The preparation process of the composite-modified asphalt was as follows [44]: DCLR was initially melted in an oven at 160 °C until it reached a fluid state. Subsequently, 500 g of 90# base asphalt was placed in an oil bath and heated to a fluid state at 170 °C. The molten DCLR was then gradually incorporated into the asphalt according to the pre-designed dosages (5%, 7%, and 9%) while keeping the temperature at 170 ± 5 °C. A high-speed shear mixer operating at 3000 r/min was employed to shear the mixture for 30 min to guarantee uniform dispersion of the DCLR. After that, SBS (at dosages of 2%, 3%, or 4%) and aromatic oil (at dosages of 2%, 4%, or 6%) were added in accordance with the specified proportions. Shearing continued at 170 °C and 2500 r/min for 45 min. Finally, the mixture was placed in an oven at 170 °C for a 2 h maturation period. During the maturation process, the mixture was stirred at a low speed every 30 min to ultimately obtain a homogeneous composite-modified asphalt sample. The preparation process of the composite-modified asphalt is shown in Figure 1.

### 2.3. Experimental Methods

#### 2.3.1. DSR Test

The Discovery HR-1 DSR was employed to systematically evaluate the alterations in the high-temperature rheological properties of the base asphalt and the nine composite-modified asphalt samples, both prior to and subsequent to aging. This evaluation was conducted through temperature sweep tests, rutting factor analysis, and MSCR tests.

(1) Temperature Sweep Test

Tests were carried out on both the original asphalt samples and those that underwent short-term aging through the Rolling Thin-Film Oven Test (RTFOT). The test was conducted under the strain-controlled mode. The temperature range was set from 46 to 82 °C at a 6 °C interval, with a strain of 1% and an angular frequency of 10 rad/s applied. A parallel plate geometry having a diameter of 25 mm and a 1 mm gap was employed. This test provided parameters such as complex modulus ($G^{*}$), phase angle (δ), and the rutting factor ($G^{*}/\sin\delta$).

(2) MSCR Test

The MSCR test was conducted on the short-term-aged asphalt samples using the DSR. The test temperatures were set at 64 °C and 70 °C, and a 15 min temperature equilibration period was implemented before the testing. The same parallel plate geometry with a diameter of 25 mm and a gap of 1 mm was employed.

The percent recovery (R) characterizes the material’s ability to recover its deformation after stress removal. The non-recoverable creep compliance ($J_{nr}$) reflects the material’s resistance to permanent deformation. The stress sensitivity index ($J_{nr\text{-}diff}$) indicates the material’s sensitivity to stress levels. The formulas for calculating R, $J_{nr}$, and $J_{nr\text{-}diff}$ are presented below:

(1) $$R=\frac{\varepsilon_{p}-\varepsilon_{u}}{\varepsilon_{p}}$$

(2) $$J_{nr}=\frac{\varepsilon_{u}}{\sigma }$$

(3) $$J_{nr\text{-}diff}=\frac{J_{nr3.2}-J_{nr0.1}}{J_{nr0.1}}$$

where:

$\varepsilon_{p}$—peak strain;

$\varepsilon_{u}$—unrecovered strain;

σ—creep loading stress.

#### 2.3.2. BBR Test

In accordance with the specifications of the American Strategic Highway Research Program (SHRP), the BBR test was employed to evaluate the low-temperature performance of asphalt. Creep stiffness (S) and creep rate (m-value) were adopted as the crucial indicators. According to the Superpave performance grading requirements, the test results at a loading time of 60 s were taken into account, with the acceptance criteria being S ≤ 300 MPa and m ≥ 0.3. In this research, BBR tests were carried out at −6 °C, −12 °C, and −18 °C to ascertain the S and m values of the asphalt binders, thus systematically evaluating their low-temperature rheological behavior. The values of S and m were calculated using the formulas presented below:

(4) $$S(t)=\frac{PL^{3}}{4bh^{3}\delta (t)}$$

(5) $$m=\frac{lg(s)}{lg(t)}$$

where:

P—constant load;

L—beam span length (mm);

b—beam width (mm);

h—beam height (mm);

δ(t)—mid-span deflection at loading time t.

#### 2.3.3. FTIR Test

The infrared spectral analyses of the base asphalt, composite-modified asphalt, and their aged samples were conducted using a Nexus-type FTIR. Prior to testing, the asphalt samples were heated in an oven at 160 °C until a flow state was reached, and then uniformly coated onto potassium bromide windows to prepare thin films of even thickness. The scanning range was set from 4000 to 400 cm^−1^, with a resolution of 4 cm^−1^, and 64 scans were performed for each sample. By comparing the variations in the positions and intensities of characteristic absorption peaks among different samples, the evolution patterns of functional groups in the asphalt before and after composite modification and during the aging process were analyzed, thereby revealing the composite modification mechanism.

#### 2.3.4. SEM Test

The microscopic morphologies of DCLR, base asphalt, and the composite-modified asphalt were examined using SEM. The samples were cryogenically fractured in liquid nitrogen, sputter-coated with gold, and then mounted on SEM specimen stubs. Imaging was carried out at an accelerating voltage of 10 kV with magnifications spanning from 500× to 5000×. The SEM micrographs were analyzed to characterize the microstructure of DCLR, evaluate the dispersion state of the modifiers within the asphalt matrix, and observe the evolution of the microstructure during the aging process. This analysis is intended to clarify the mechanisms underlying the performance changes in the composite-modified asphalt at the microscopic level.

## 3. Results and Discussion

### 3.1. Analysis of Temperature Sweep Results

- Complex Shear Modulus

**Figure 2:**
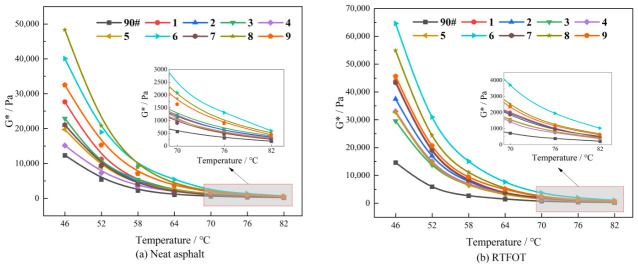
Variation in complex modulus with temperature for samples in different states.

Figure 2 illustrates the variation in the complex modulus with temperature for different asphalt samples before and after aging. The analysis reveals the following:

(1) The complex modulus exhibits a decreasing trend as temperature rises, gradually approaching zero at high temperatures. This is mainly due to the enhanced thermal motion of asphalt molecules and the weakening of intermolecular interactions, which decreases the material’s resistance to deformation.

(2) For the original (unaged) asphalt samples, the complex modulus values in descending order are: Sample 8 > 6 > 9 > 1 > 3 > 7 > 2 > 5 > 4 > base asphalt. The curve of the base asphalt always stays at the lowest position, which indicates that DCLR composite modification enhances the high-temperature deformation resistance of asphalt to varying degrees.

(3) Following short-term aging, the asphalt samples exhibit a similar pattern of change in complex modulus. This pattern is characterized by an initial rapid decrease, followed by stabilization as the temperature rises. In comparison with the unaged samples, the complex modulus of the composite-modified asphalt generally increases after aging, which indicates material hardening. This trend is consistent with the results of the 25 °C penetration test. The hardening primarily results from oxidation reactions during aging. These reactions generate polar functional groups (e.g., carbonyl and sulfoxide groups), thus enhancing intermolecular van der Waals forces and improving the resistance to external loads.

(4) Within the temperature range of 46–82 °C, the maximum complex modulus of the short-term-aged, composite-modified asphalt is higher than that of the unaged composite-modified asphalt, while the minimum complex modulus is lower. This indicates that short-term aging has a significant impact on the complex modulus of the composite-modified asphalt.

(5) As the temperature rises, the complex modulus of the base asphalt decreases to zero at a significantly faster rate compared to that of the composite-modified asphalt. This indicates that the base asphalt has a higher temperature susceptibility.

(6) Compared with the system containing only DCLR [44], the complex modulus values of the composite-modified asphalt in this study were increased by approximately 50–120%, which was found to be not significantly different from the DCLR + SBS binary system reported in the literature [25]. Therefore, it was demonstrated that the introduction of SBS was the key to substantially enhancing the high-temperature deformation resistance.

b. Phase Angle

**Figure 3:**
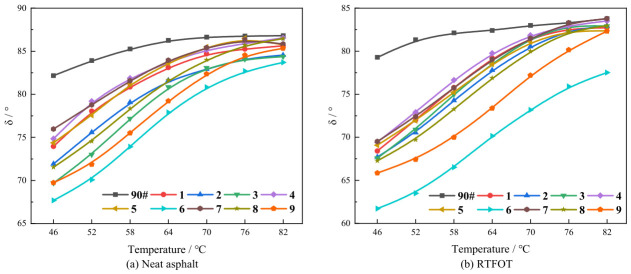
Variation in phase angle with temperature for samples in different states.

Figure 3 illustrates the variation in the phase angle with temperature for asphalt samples under different conditions, leading to the following conclusions:

(1) For both the original and the RTFOT short-term-aged composite-modified asphalt samples, the phase angle gradually increases as the temperature rises. This indicates a reduction in the proportion of the elastic component within the material, suggesting a shift towards a viscous-dominated flow state. When the phase angle nears 90°, the asphalt exhibits predominantly viscous flow behavior, with its elastic recovery capacity significantly reduced.

(2) At the same temperatures, the phase angle of the asphalt after short-term aging is lower than that of the original samples. This indicates an improvement in the elastic properties and a better resistance to deformation after aging. This phenomenon is mainly due to the transformation of saturates and aromatics into resins and asphaltenes during the aging process, which reduces the viscous components and increases the elastic components of the asphalt. Meanwhile, the increase in aromaticity and condensation indices enhances the material’s stiffness, jointly leading to the observed decrease in the phase angle.

(3) At the same temperature, the phase angle of the base asphalt is consistently higher than that of the composite-modified asphalt. This phenomenon can be attributed to the fact that the DCLR composite-modified asphalt contains a high proportion of heavy components, particularly asphaltenes, which impart superior elastic characteristics to it. In summary, as the temperature increases, the complex modulus of asphalt decreases, while the phase angle increases. This indicates a transition in the material’s behavior from being predominantly elastic to predominantly viscous. Under high-temperature conditions, the complex modulus of the base asphalt drops rapidly, and its phase angle increases sharply. This leads to a significant reduction in deformation resistance and a higher susceptibility to distresses such as rutting. In contrast, the composite-modified asphalt maintains a relatively higher complex modulus and a relatively lower phase angle across the entire investigated temperature range, which demonstrates a remarkable improvement in its high-temperature performance.

(4) Related studies have indicated [25,44] that for a modified asphalt containing 10% DCLR, the phase angle at 64 °C was decreased only from approximately 86.5° to about 83.2°, corresponding to a reduction of approximately 3.3°. In contrast, the reduction in phase angle for the ternary system in this study exceeded 10°, suggesting that the synergistic introduction of SBS and aromatic oil greatly enhanced the elastic response of the asphalt. Even when compared with the DCLR + SBS binary system reported in the literature, the optimal formulation in this study exhibited an even lower phase angle, demonstrating that the combination of a high DCLR content with SBS and aromatic oil can further optimize the viscoelastic balance.

### 3.2. Analysis of High-Temperature Rutting Factor

**Figure 4:**
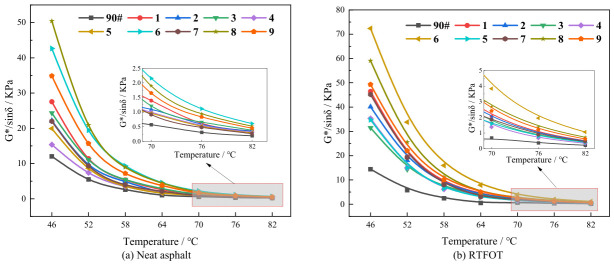
Variation in rutting factor with temperature for samples in different states.

Figure 4 presents the variation in the rutting factor with temperature for asphalt samples under different conditions. The analysis of the experimental results is as follows:

(1) As the temperature rises, the $G^{*}/\sin\delta$ of all asphalt samples exhibits a pattern of initially experiencing a rapid decline and then gradually stabilizing, which aligns with the observed trend of the complex modulus. This phenomenon takes place because the increasing temperature weakens the intermolecular forces within the asphalt. Consequently, the viscous components increase substantially, while the elastic components decrease accordingly. This results in a more pronounced viscous flow behavior at high temperatures and a subsequent reduction in resistance to permanent deformation.

(2) Within the higher temperature range, the ranking of rutting factor values for the original asphalt samples is as follows: Sample 8 > 6 > 9 > 1 > 3 > 2 > 7 > 5 > 4 > base asphalt. After short-term aging, the ranking changes to: Sample 6 > 8 > 9 > 1 > 7 > 2 > 4 > 5 > 3 > base asphalt. The comparison indicates that Samples 6 and 8 consistently demonstrate superior high-temperature performance both before and after aging. This suggests that the composite modifiers can effectively increase the asphalt’s viscosity, inhibit high-temperature flow deformation, and thereby enhance the rutting resistance.

(3) After RTFOT short-term aging, the $G^{*}/\sin\delta$ values of all asphalt samples increased significantly. This is mainly due to the transformation of light components into asphaltenes during the aging process, which enhances the asphalt’s consistency and stiffness. Meanwhile, cross-linking and polymerization of small molecules take place, resulting in the formation of structures with higher molecular weights. This reinforces the intermolecular bonding within the asphalt, thereby improving the rutting resistance of the composite-modified asphalt.

(4) Related studies have indicated [25,44] that for a modified asphalt containing 10% DCLR, the $G^{*}/\sin\delta$ at 64 °C was approximately 3.8 kPa; for the DCLR + SBS binary-modified asphalt, the $G^{*}/\sin\delta$ at the same temperature was approximately 8.7 kPa. In contrast, a higher rutting factor was obtained for Samples No. 6 and No. 8 in this study at similar or even lower total modifier contents, indicating that the ternary system of “DCLR + SBS + aromatic oil” exhibited a superior synergistic enhancement effect. In particular, the addition of aromatic oil not only improved the low-temperature performance but also, by promoting the uniform dispersion of DCLR and SBS, avoided local stress concentration caused by the agglomeration of either component, thereby further unleashing the network-reinforcement efficiency of SBS.

### 3.3. Analysis of MSCR Test Results

- Creep Recovery Rate

**Figure 5:**
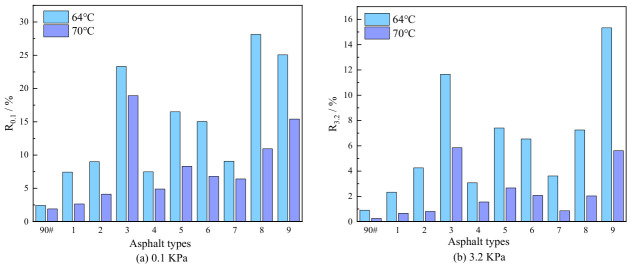
Variation in creep recovery rate for various asphalt binders under two stress levels.

Figure 5 illustrates the variation in the creep recovery rate for different asphalt binders under two stress levels. The analysis yields the following conclusions:

(1) Under various stress conditions, the creep recovery rate of the composite-modified asphalt is notably higher than that of the base asphalt. Taking the results at 64 °C as an illustration, under a stress of 0.1 kPa, the descending order of $R_{0.1}$ values is as follows: Sample 8 > 9 > 3 > 5 > 6 > 7 > 2 > 4 > 1 > base asphalt. Their $R_{0.1}$ values are 11.53, 10.27, 9.56, 6.76, 6.16, 3.72, 3.69, 3.07, and 3.05 times that of the base asphalt, respectively. Under a stress of 3.2 kPa, the order is altered to: Sample 9 > 3 > 5 > 8 > 6 > 4 > 2 > 7 > 1 > base asphalt. The base asphalt exhibits the lowest recovery rate under higher stress, which indicates its limited ability to resist load-induced deformation. The higher R values of the composite-modified asphalt suggest that the modifiers have modified the viscoelastic composition of the asphalt, improving its delayed elastic recovery capacity and reducing viscous deformation. As a result, the composite asphalt shows better high-temperature deformation resistance under load, which is consistent with the “enhanced elasticity and reduced viscosity” trend reflected by the decreased phase angle in the temperature sweep tests.

(2) As the stress level increases, the creep recovery rate for all asphalt binders shows a declining trend. Under actual pavement conditions, heavier wheel loads can lead to more pronounced rutting and permanent deformation. For example, at 64 °C, when the stress increases from 0.1 kPa to 3.2 kPa, the R value of the base asphalt decreases from 2.44 to 0.91, which indicates that it has poor elastic recovery capacity and is prone to damage under high stress. The decreasing order of R values for the composite-modified asphalt are as follows: Sample 8 > 3 > 9 > 5 > 6 > 7 > 1 > 2 > 4, which shows that high stress can significantly undermine their creep recovery performance and deformation resistance.

(3) An increase in temperature also causes a decrease in the creep recovery rate of asphalt. This is because the asphalt has a higher temperature susceptibility; when the temperature rises, the sample softens, leading to a smaller proportion of recoverable strain during the unloading recovery phase. Consequently, the R value decreases as the temperature rises.

b. Non-Recoverable Creep Compliance

**Figure 6:**
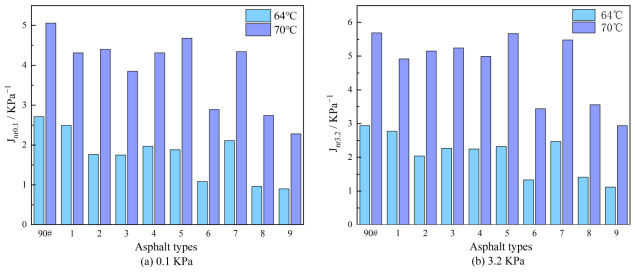
Variation in non-recoverable creep compliance for various asphalt binders under two stress levels.

While the R effectively reflects the elastic deformation capacity of composite-modified asphalt, the extent of permanent deformation is more directly characterized by the non-recoverable creep compliance. Figure 6 illustrates the variation of $J_{nr}$ for the different asphalt binders under two stress levels, with the results as follows:

(1) Under different stress levels, the $J_{nr}$ values of the composite-modified asphalt are lower than those of the base asphalt. Taking the results at 64 °C as an example, under a stress of 0.1 kPa, the order of $J_{nr0.1}$ values from highest to lowest is: Base asphalt > Sample 1 > 7 > 4 > 5 > 2 > 3 > 6 > 8 > 9. Under a stress of 3.2 kPa, the order of $J_{nr3.2}$ is: Base asphalt > Sample 1 > 7 > 5 > 3 > 4 > 2 > 8 > 6 > 9. This indicates that the composite-modified asphalt possesses a higher proportion of elastic components and greater resistance to viscous flow deformation at high temperatures, resulting in significantly superior rutting resistance compared to the base asphalt. The overall ranking of high-temperature performance is: Sample 9 > 6 > 8 > 2 > 4 > 3 > 5 > 7 > 1 > base asphalt.

(2) An increase in stress level generally leads to an increase in $J_{nr}$ for all asphalt binders. At 64 °C, when the stress increases from 0.1 kPa to 3.2 kPa, the $J_{nr}$ of the base asphalt rises from 2.71 to 2.94, representing a relative increase of 8.49%. In contrast, the relative increases for the composite-modified asphalt samples are 11.24%, 15.91%, 29.71%, 14.21%, 23.40%, 23.15%, 17.06%, 46.88%, and 24.44%, respectively. This suggests that the composite-modified asphalt is more sensitive to stress variation, and its modified system exhibits more pronounced viscoelastic adjustment behavior under applied stress.

(3) As both temperature and stress increase, the $J_{nr}$ values of all asphalt binders show an upward trend. This indicates that high temperature and heavy loading have a similar detrimental effect on the high-temperature deformation resistance of asphalt.

(4) Compared with the system containing only DCLR [44], the $J_{nr}$ values of the composite-modified asphalt in this study (e.g., Sample No. 3, No. 8, and No. 9) were reduced by more than 52%, indicating that the introduction of SBS was identified as the key to substantially improving the permanent deformation resistance. When compared with the DCLR + SBS binary system reported in the literature [25], Sample No. 9 in this study achieved a comparable or even lower $J_{nr}$ value (0.58 kPa^−1^), while simultaneously attaining superior low-temperature performance.

c. Percentage Difference in Non-Recoverable Creep Compliance

**Figure 7:**
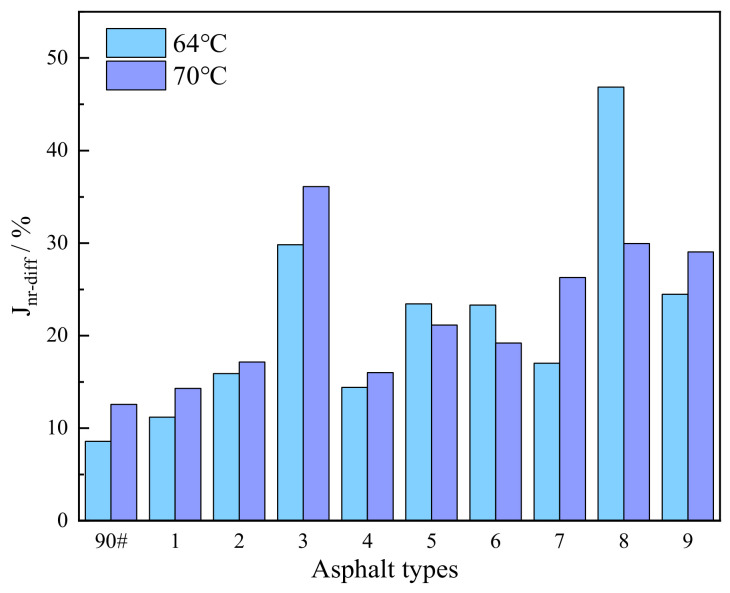
Variation in the percentage difference in non-recoverable creep compliance for various asphalt binders.

Asphalt pavements are subjected to complex axial loading during service. The percentage difference in $J_{nr-diff}$ serves as a key indicator for evaluating the material’s sensitivity to load variation: a lower $J_{nr-diff}$ value signifies reduced sensitivity of the asphalt to changes in load, which is beneficial for extending pavement service life. Figure 7 presents the variation in $J_{nr-diff}$ for the different asphalt binders. Taking the results at 64 °C as an example, the order of $J_{nr-diff}$ values from highest to lowest is: Sample 8 > 3 > 9 > 5 > 6 > 7 > 2 > 4 > 1 > base asphalt. This result indicates that the composite-modified asphalt exhibits lower stress sensitivity, which contributes to enhancing the durability of asphalt pavements under heavy traffic conditions.

### 3.4. Analysis of BBR Test Results

**Figure 8:**
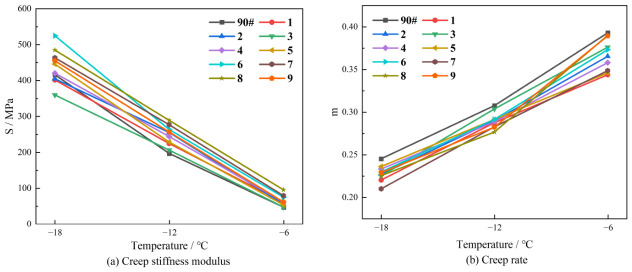
Variation in stiffness modulus and creep rate with temperature for various asphalt binders.

Figure 8 illustrates the variation trends of S and m-value with temperature for the various asphalt binders. It is evident that the S values for both the base asphalt and the nine composite-modified asphalt samples increase as the temperature decreases, while the m-values show a corresponding decline. This indicates a reduction in deformation capacity and an increase in brittleness of the asphalt under low-temperature conditions. This phenomenon occurs because, as the temperature drops, the asphalt gradually approaches its glass transition region. The mobility of molecular chain segments decreases, leading to a significant increase in material rigidity. Consequently, the stress relaxation capability of the asphalt deteriorates at low temperatures.

In most DCLR-modified systems reported in the literature [44], the m-value at −12 °C was generally found to be below 0.25, thus failing to meet the Superpave criterion (m ≥ 0.3). In contrast, through the compatibilizing effect of aromatic oil, Sample No. 3 (m = 0.312) and Sample No. 9 (m = 0.298) in this study enabled, for the first time, the low-temperature performance of high-content DCLR-modified asphalt to approach or satisfy the specification requirements. This confirmed the effectiveness of the ternary synergistic strategy—namely, “DCLR provides high-temperature performance, SBS provides elastic recovery, and aromatic oil improves low-temperature compatibility”—which represented a fundamental advancement over the binary systems reported in the literature.

### 3.5. Analysis of FTIR Test Results

**Figure 9:**
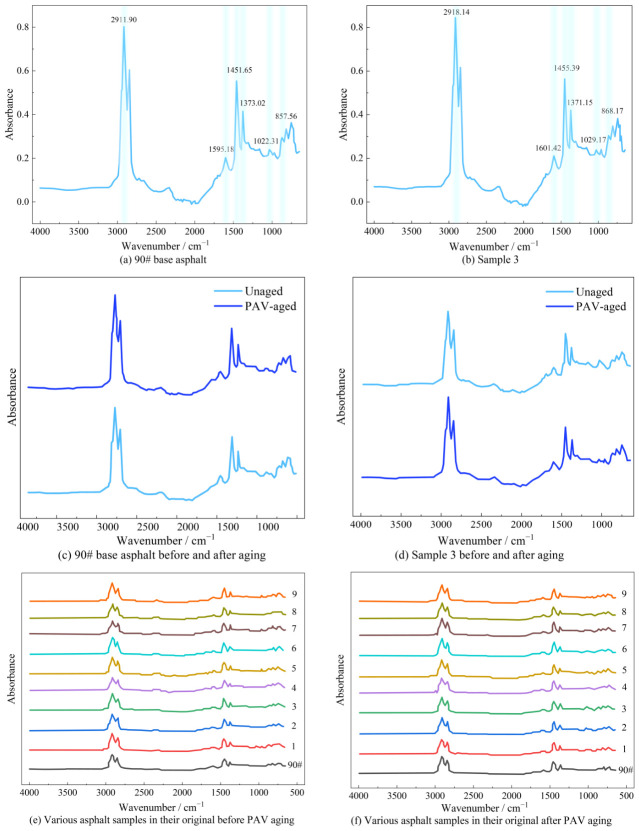
Absorbance changes in various asphalts before and after aging.

The FTIR analysis was performed using the base asphalt and the No. 3 modified asphalt as examples. The infrared absorption curves before and after aging were plotted to analyze the molecular structure and functional groups of the asphalt, as well as to investigate the compositional changes in the asphalt after long-term aging. The infrared spectra of each asphalt before and after aging are presented in Figure 9, and the functional group distributions of the base asphalt and the No. 3 composite-modified asphalt are summarized in Table 6. The analysis is as follows:

(1) The absorption spectra of all asphalt samples exhibited similar shapes, and the positions and intensities of the absorption peaks were almost identical. This result indicates that, for the composite-modified asphalt, the composite modifier and the base asphalt underwent physical blending, with no generation of new chemical bonds or functional groups.

(2) After aging, no new absorption peaks were observed in the composite-modified asphalt, suggesting that the composite modifier could not alter the internal molecular structure of the asphalt, and only physical reactions occurred during the aging process.

### 3.6. Analysis of SEM Test Results

**Figure 10:**
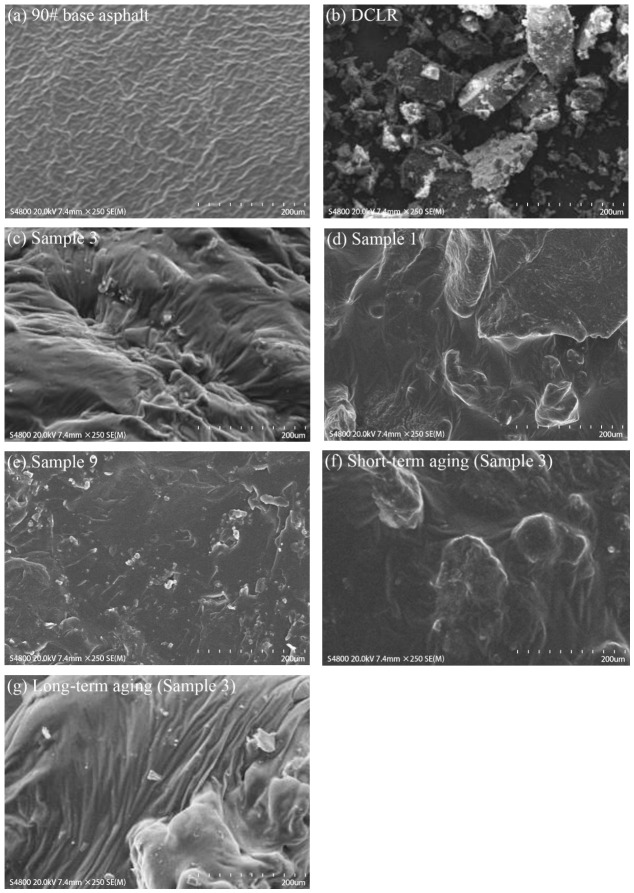
SEM images for various asphalt binders.

In this SEM analysis, Sample No. 3 (5% DCLR, 4% SBS, and 6% aromatic oil), which exhibited the most outstanding low-temperature performance, was taken as an example. By comparing the micromorphology of the base asphalt, Sample No. 1 (5% DCLR, 2% SBS, and 2% aromatic oil), Sample No. 9 (9% DCLR, 4% SBS, and 4% aromatic oil), and Sample No. 3, as well as their evolutions during the aging process, it was aimed to directly establish the intrinsic relationship between the low-temperature rheological properties and the microstructure. Figure 10 presents the SEM observations. The analysis reveals the following:

(1) The DCLR presents a porous, cross-linked microstructure on its surface, which can be ascribed to the volatilization and reaction of liquefiable components during the direct coal liquefaction process.

(2) The surface of the base asphalt appeared overall smooth and homogeneous, with sporadic impurities present locally. In Sample No. 1, a significant agglomeration of DCLR particles was observed, accompanied by non-uniform distribution and weak interfacial bonding, which led to stress concentration at low temperatures and the absence of an elastic network at high temperatures. Rheologically, this was manifested by an R of only 2.5% under the condition of 64 °C and 0.1 kPa, a high $J_{nr}$ of 1.85 kPa^−1^, and an m-value of only 0.28 at a low temperature of −12 °C, failing to meet the Superpave criterion. In Sample No. 9, high-content DCLR particles were densely distributed within the SBS network, forming a compact filling structure that endowed the material with the highest elastic modulus and permanent deformation resistance (R = 28.7%, $J_{nr}$ = 0.58 kPa^−1^, indicating optimal high-temperature performance). However, due to a relatively insufficient amount of aromatic oil, the interfacial flexibility and low-temperature molecular chain mobility were slightly compromised (with an m-value of 0.298 at −12 °C), marginally below the specification limit. For Sample No. 3, DCLR particles were uniformly dispersed, SBS formed a continuous elastic network, and aromatic oil effectively acted as a compatibilizer. The microscopic configuration was characterized as “physical reinforcement by DCLR, elastic network by SBS, and bridging by aromatic oil”, which endowed it with good high-temperature deformation recovery capacity (R = 9.56%) and optimal low-temperature stress relaxation (with an m-value of 0.312 at −12 °C), fully satisfying the specification requirements. It was thus revealed that an increase in SBS content significantly improved dispersion and elastic recovery, thereby enhancing low-temperature performance; an increase in DCLR content substantially strengthened the high-temperature deformation resistance, but a sufficient amount of aromatic oil was required to maintain low-temperature cracking resistance. A balanced proportion of the three components was identified as the key to achieving both high-and low-temperature performance.

(3) In Sample 3 (the composite-modified asphalt), a large number of DCLR particles and elongated SBS chains are distributed. After short-term aging, the quantity of discrete modifier particles decreases, and large molecular aggregates, like clustered gel-like substances, emerge. After long-term aging, the particles are further diminished, resulting in the formation of bulk particulate structures. This indicates that during thermos-oxidative aging, the composite-modified asphalt system gradually forms complex macromolecular micelles. The evolution of this microstructure is consistent with macroscopic aging behaviors such as hardening and agglomeration.

In summary, the improvement of the high-and low-temperature performance of the composite-modified asphalt is closely associated with the uniformity of its microstructure, interfacial compatibility, and structural densification during the aging process. Taking Sample No. 3 composite-modified asphalt as an illustration, its excellent low-temperature performance stems from the favorable compatibility facilitated by the plasticizing effect of aromatic oil and the continuous elastic network constructed by SBS. Its superior high-temperature performance is attributed to the physical reinforcement and network anchoring effect provided by the uniformly dispersed DCLR particles. At the microscopic level, both systems feature uniformly dispersed modifiers and strong interfacial bonding, which lay the structural foundation for their remarkable macroscopic rheological performance. The intrinsic relationship among structure, performance, and durability in the DCLR–SBS–aromatic oil ternary composite modification system offers solid micromechanical evidence for the rational design of high-performance modified asphalt.

## 4. Conclusions

In this study, the high-and low-temperature rheological properties and microstructural evolution of the DCLR–SBS–aromatic oil ternary composite-modified asphalt before and after aging were systematically evaluated through temperature sweep, MSCR, BBR, FTIR, and SEM tests. The main conclusions are as follows.

(1) A significant improvement in high-temperature performance was observed, and the degree of improvement was positively correlated with the DCLR and SBS contents. The $G^{*}$ of the composite-modified asphalt at 82 °C was increased by approximately 2.5 times compared to that of the base asphalt, with Sample 8 (9% DCLR, 3% SBS, and 2% aromatic oil) exhibiting the highest $G^{*}$ value after aging. In terms of the $G^{*}/\sin\delta$ at 70 °C, Sample 6 (7% DCLR, 4% SBS, and 2% aromatic oil) and Sample 8 reached 12.5 kPa and 11.8 kPa, respectively, which were more than three times that of the base asphalt. The MSCR test further revealed that, under the conditions of 64 °C and 0.1 kPa, the R of Sample 9 (9% DCLR, 4% SBS, and 6% aromatic oil) was as high as 28.7%, which was 11.5 times that of the base asphalt, while its non-recoverable creep compliance $J_{nr}$ was as low as 0.58 kPa^−1^, representing a reduction of 78.6% compared to the base asphalt. Mechanistically, the high asphaltene components in DCLR increased the consistency and elasticity of the asphalt, whereas the three-dimensional network structure formed by SBS endowed the material with excellent deformation recovery capability. The synergistic effect of the two components significantly suppressed permanent deformation at high temperatures.

(2) A noticeable improvement in low-temperature performance was achieved, with aromatic oil being the key compatibilizing factor. The BBR test results showed that, at –12 °C, Sample 3 (5% DCLR, 4% SBS, and 6% aromatic oil) exhibited an m-value of 0.312 and an S of 218 MPa, fully meeting the Superpave criteria (m ≥ 0.3, S ≤ 300 MPa). Sample 9 had an m-value of 0.298, which also approached the specification requirement. Mechanistically, a high dosage of aromatic oil (6%) acted as a compatibilizer, effectively improving the interfacial compatibility between DCLR and the base asphalt, reducing the degree of DCLR particle agglomeration, and enabling the modifiers to form a uniform and continuous distribution in the asphalt. Consequently, stress concentration at low temperatures was alleviated, and the stress-relaxation ability of the asphalt was enhanced. In contrast, for formulations with excessively high DCLR and SBS contents but insufficient aromatic oil (e.g., Sample 8), although excellent high-temperature performance was obtained, the low-temperature m-value decreased to below 0.28, indicating that the balance among the three components is critical for achieving both high-and low-temperature performance.

(3) The composite modification was dominated by physical blending, and the aging process was accompanied by physical structure rearrangement rather than chemical changes. FTIR analysis revealed that no new characteristic absorption peaks appeared in any of the composite-modified asphalts either before or after modification or aging, indicating that no significant chemical reaction occurred between the modifiers and the base asphalt. After aging, the light components in the asphalt were reduced, while the relative contents of resins and asphaltenes increased; however, no new functional groups were generated. SEM observations showed that, after short-term aging, agglomerates of gel-like substances appeared around the modifier particles, which further evolved into dense macromolecular micelle structures after long-term aging. This physical densification process was highly consistent with the macroscopic aging behaviors of increased hardness and decreased ductility.

(4) The synergistic mechanism of the three components was clarified, and the formulation should be selected based on performance requirements. A comprehensive comparison of the nine formulations revealed the following: if high-temperature rutting resistance is the primary objective, Sample 8 or Sample 6 (high DCLR with medium-to-high SBS, and not excessively high aromatic oil) is recommended; if low-temperature crack resistance is the primary objective, Sample 3 (moderate DCLR, high SBS, and high aromatic oil) is recommended; for a balance of both high-and low-temperature performance, Sample 9 (high DCLR, high SBS, and high aromatic oil) achieves the best trade-off, with the lowest $J_{nr}$ value at high temperatures and an m-value close to 0.30 at low temperatures. The ternary synergistic mechanism revealed in this study—namely, “DCLR enhances high-temperature performance, SBS provides elastic recovery, and aromatic oil improves compatibility”—provides a clear theoretical basis and technical support for the high-value and high-performance application of DCLR in asphalt pavements.

## Figures and Tables

**Table 1:** Technical specifications of the 90# base asphalt.

Test Item	Unit	Technical Requirement	Test Result
Penetration (25 °C, 100 g, 5 s)	0.1 mm	80~100	82.6
Softening point (ring & ball)	℃	≥43	52.5
Dynamic viscosity (60 °C)	Pa·s	≥140	157
Ductility (10 °C)	cm	≥20	34.0
Relative density (15 °C)	g/cm^3^	Measured	1.031
Residue after TFOT (or RTFOT)			
Mass change	%	≤±0.8	0.25
Retained penetration ratio (25 °C)	%	≥57	65.4
Retained ductility (10 °C)	cm	≥8	8.2

**Table 2:** Properties of DCLR.

Technical Indicator	Density (g/cm^3^)	Penetration at 25 °C (0.1 mm)	Ductility at 10 °C (cm)	Softening Point (°C)
Test result	1.24	4.8	1.8	175

**Table 3:** Basic properties of SBS.

Grade	Structure	Block Ratio (S/B)	Volatile Content (%)	Ash Content (%)	Tensile Strength (MPa)	Elongation at Break (%)	Permanent Set (%)	Shore A Hardness	Melt Flow Rate (g/10 min)
YH-791	Linear	30/70	≤0.7	≤0.2	≥15	≥700	≤40	68	0.5~2.5

**Table 4:** Factors and levels for composite-modified asphalt.

Level	DCLR Content (%)	SBS Content (%)	Aromatic Oil Content (%)
1	5	2	2
2	7	3	4
3	9	4	6

**Table 5:** Orthogonal test plan for composite-modified asphalt.

Group No.	DCLR Content (%)	SBS Content (%)	Aromatic Oil Content (%)
1	5	2	2
2	5	3	4
3	5	4	6
4	7	2	4
5	7	3	6
6	7	4	2
7	9	2	6
8	9	3	2
9	9	4	4

**Table 6:** Functional group distribution of the base asphalt and the No. 3 composite-modified asphalt.

90# Base Asphalt	Sample 3	Peak Assignments
2911.90	2918.14	Symmetric and asymmetric stretching vibrations of methylene (-CH_2_-)
1595.18	1601.42	C=C (benzene ring skeleton vibration) and C=O absorption
1451.65	1455.39	In-plane C–H stretching vibrations of C–CH_3_ and CH_2_
1373.02	1371.15	Scissor vibration absorption of methyl (-CH_3_-)
1022.31	1029.17	Stretching vibration of the sulfoxide group (S=O)
857.56	868.17	Out-of-plane vibration of = C-H on the benzene ring

## Data Availability

All data generated or used during the study appear in the submitted article.
